# Centralizing the Knowledge and Interpretation of Pain in Chemotherapy-Induced Peripheral Neuropathy: A Paradigm Shift towards Brain-Centric Approaches

**DOI:** 10.3390/brainsci14070659

**Published:** 2024-06-28

**Authors:** Mário Cunha, Isaura Tavares, José Tiago Costa-Pereira

**Affiliations:** 1 Department of Biomedicine, Unit of Experimental Biology, Faculty of Medicine, University of Porto, Alameda Prof. Hernâni Monteiro, 4200-319 Porto, Portugal; mariocunha454@gmail.com (M.C.); jcostapereira@fcna.up.pt (J.T.C.-P.); 2 I3S—Institute of Investigation and Innovation in Health, University of Porto, Rua Alfredo Allen 208, 4200-135 Porto, Portugal; 3 Faculty of Nutrition and Food Sciences, University of Porto, Rua do Campo Alegre 823, 4150-180 Porto, Portugal

**Keywords:** pain modulation, noradrenaline, serotonin, antidepressants, imaging studies

## Abstract

Chemotherapy-induced peripheral neuropathy (CIPN) is a side effect of cancer treatment, often linked with pain complaints. Patients report mechanical and thermal hypersensitivity that may emerge during chemotherapy treatment and may persist after cancer remission. Whereas the latter situation disturbs the quality of life, life itself may be endangered by the appearance of CIPN during cancer treatment. The causes of CIPN have almost entirely been ascribed to the neurotoxicity of chemotherapeutic drugs in the peripheral nervous system. However, the central consequences of peripheral neuropathy are starting to be unraveled, namely in the supraspinal pain modulatory system. Based on our interests and experience in the field, we undertook a review of the brain-centered alterations that may underpin pain in CIPN. The changes in the descending pain modulation in CIPN models along with the functional and connectivity abnormalities in the brain of CIPN patients are analyzed. A translational analysis of preclinical findings about descending pain regulation during CIPN is reviewed considering the main neurochemical systems (serotoninergic and noradrenergic) targeted in CIPN management in patients, namely by antidepressants. In conclusion, this review highlights the importance of studying supraspinal areas involved in descending pain modulation to understand the pathophysiology of CIPN, which will probably allow a more personalized and effective CIPN treatment in the future.

## 1. Introduction

Better methods in cancer detection and the increased success of cancer treatment have led to a growing number of patients undergoing chemotherapy and an increased number of cancer survivors. Among the challenges of cancer treatment, chemotherapy-induced peripheral neuropathy (CIPN) has emerged as a significant and debilitating side effect, which may occur during cancer treatment and persist after cancer remission [1,2]. The prevalence of CIPN after chemotherapy ranges from 30% to 68% with significant variability in severity among individuals [2]. In comparison with patients that do not develop CIPN, on average patients with CIPN represent an additional demand on health services since they have the need for 12 additional outpatient visits, 3 additional days in hospital, and incur an extra USD 17,000 in medical expenses [3]. This highlights the profound impact of CIPN on the patients’ physical, social, emotional, functional, financial, and occupational well-being. The probability of developing CIPN is related to the type of chemotherapeutic drug and treatment protocol, namely the cumulative doses [2,4,5]. CIPN results from peripheral nerve injury caused by chemotherapy drugs, since it has the ability to damage peripheral nerve fibers, especially those involved in sensory and autonomic functions [6]. Small-diameter nerve fibers, unmyelinated or thin myelinated fibers responsible for conveying nociceptive information, are particularly susceptible to chemotherapeutic agents [7]. While extensive research has been conducted on the impact of CIPN on peripheral nerves [8,9], recent studies have begun to explore the consequences of peripheral damage on the spinal cord and supraspinal structures, specifically those involved in top-down pain modulation. 

The descending pain modulatory system is a network of intrinsically connected brain areas that exerts a bidirectional balance (inhibition or facilitation) of the nociceptive inputs arising from the spinal cord [10,11,12]. Despite the neurochemical complexity of the system, the neurotransmitters released at the spinal cord during top-down modulation are mainly monoamines and opioids [13]. The proper functioning of the descending pain modulatory systems is essential to maintain the balance between inhibition and facilitation. Neuroplastic changes in the descending modulatory pathways are well documented in both human and animal models of neuropathic pain [14]. These changes disrupt the balance exerted by descending modulatory circuits, enhancing facilitation over inhibition [15,16]. That imbalance has been proposed to account for the development and persistence of neuropathic pain, namely of traumatic origin [15,16]. The role of brain-mediated pain modulation has been much less addressed in CIPN; despite the dramatically growing prevalence, this is a condition due to the increased number of patients undergoing chemotherapy. In clinical settings, patients with CIPN are often treated with antidepressants which raise the levels of serotonin (5-HT)/noradrenaline (NA) in the central nervous system (CNS) [7,17]. However, the mechanisms underlying the use of those drugs in CIPN are just starting to be unraveled. Furthermore, advanced neuroimaging tools available for both preclinical and clinical models, are important translational tools [18]. Filling the information gap between preclinical and clinical studies offers an opportunity to fundamentally change our understanding of CIPN by investigating the role of the CNS in its pathogenesis and developing novel treatment approaches.

This narrative review provides an overview of the central mechanisms of CIPN involving descending pain modulation, which can leverage the approach to managing and potentially preventing this condition. 

## 2. Clinical Syndrome

In recent years, the number of cancer patients is increasing due to improved detection and better treatment of malignant conditions [19]. Cancer is now viewed as a chronic condition. While longer survival is a positive shift, it presents new challenges. Cancer survivors often deal with treatment side effects, like fatigue, emotional distress, and pain, in their post-treatment phase [1].

Chemotherapy drugs can harm the nervous system and lead to various types of neuropathies, including sensory, motor, and autonomic, affecting both large and small nerve fibers [20]. CIPN is a medical condition that refers to the damage or dysfunction of the peripheral nerves caused by chemotherapy [21]. The prevalence and incidence of CIPN vary widely depending on several factors, including the specific chemotherapy drugs used, the cumulative dose administered, and the individual characteristics of the patients [22]. Nevertheless, the individual features which may prompt a cancer patient to be more prone to develop CIPN have not been investigated in detail, and, herein, prevention is still not possible. Symptoms of CIPN can be acute or chronic. Acutely, CIPN occurs in 68% of patients and, if severe enough, it can require a reduction in the dose of chemotherapy or even stopping it before completing the planned protocol [2,4]. The prevalence of CIPN is estimated to be 68% in the first month, 60% after 3 months, and 30% after 6 months [2,4]. Since specific chemotherapeutic agents (e.g., cisplatin, paclitaxel) remain in the body for a long period [23], some patients experience paradoxical worsening and/or intensification of symptoms even after finishing the chemotherapy protocol, a phenomenon known as “coasting” [24,25]. This condition increases the financial burden on healthcare systems, prevents patients from working, and has a huge impact on the quality of life of cancer survivors [3,22].

The clinical presentation of CIPN varies greatly according to the type of chemotherapeutic drug, along with its dose, but factors such as the duration of chemotherapy treatment and assessment method are also important [26]. Sensory symptoms are the most common, but motor weakness, autonomic dysfunction, and cranial nerve involvement may occur [2,27]. Patients often describe sensory symptoms such as tingling, numbness, pressure, and paresthesia induced by touching warm or cool objects in a symmetric “glove and stocking” neuropathy pattern [28]. Although pain is not always a presenting feature [29], painful sensations, including spontaneous burning, shooting or electric shock-like pain, and mechanical or thermal allodynia/hyperalgesia, frequently occur [30]. In more severe cases, vibration sense and proprioception may be lost, which can significantly impact daily functioning [24]. Motor symptoms, although less common than sensory symptoms, also have severe consequences. They typically consist of weakness in the extremities, difficulties with gait and balance, and impaired coordination and have a significant, although sometimes underestimated, influence on the patient’s quality of life. For instance, cancer patients who experience CIPN are at a three-fold higher risk of experiencing falls [31]. In severe cases, CIPN can lead to paresis and complete patient immobilization [32]. Fine motor difficulties are also reported and patients may encounter challenges with daily tasks like buttoning clothing [24]. Autonomic symptoms are infrequent and the main features are orthostatic hypotension, altered sexual or urinary function, and constipation [27,32]. 

## 3. Clinical Assessment

Clinically, there is no uniform approach or standard tools for CIPN assessment [33,34]. In the context of painful CIPN, screening questionnaires can be used as a standard method for assessing and diagnosing neuropathic pain [35,36]. Other specific questionnaires commonly utilized are the National Cancer Institute (NCI-CTC) and the Total Neuropathy Score (TNS) [4]. These assessments help classify CIPN into grades or stages and provide a basis for tracking symptom changes over time [34]. The European Organization for Research and Treatment of Cancer (EORT) published the EORT-CIPN 20 guidelines that have proven valuable in clinical practice and research settings, particularly in the context of large oncology clinical trials [34]. 

Nerve conduction studies and electromyography can be useful in diagnosing CIPN. Those parameters can be especially valuable in cases where clinical symptoms may not fully reflect the extent of nerve damage, offering a more in-depth evaluation [37]. Similarly to other neuropathies, conduction nerve studies are considered the gold standard for detecting and monitoring CIPN [38]. However, these methods are expensive and time consuming and thus have had limited use in clinical settings [39]. Therefore, current CIPN assessment strategies are not sensitive or practical for regular clinical monitoring, making it difficult to predict who is at high risk for harmful side effects or irreversible damage [40]. 

## 4. Management of CIPN

As in other clinical conditions, research in CIPN can be conducted with the goals of prevention and/or treatment. To date, there are no proven ways to prevent CIPN and effective treatments are limited. For many patients, when the signs of CIPN first appear, reduction of chemotherapy or even its cessation can be implemented [41], which may negatively affect the overall survival of cancer patients [42]. Determining whether CIPN can be mitigated or prevented without compromising life-saving chemotherapy is crucial. Currently, managing CIPN poses significant challenges. Firstly, predicting who will develop CIPN is difficult. Secondly, symptoms can emerge at any stage during chemotherapy. Thirdly, there are no approved drugs for the effective prevention of CIPN. Finally, while duloxetine is the only approved drug for treating CIPN, its effectiveness is moderate [41,43]. Curiously, despite the expectation that pain control from the brain would be a target in CIPN studies—given that variations in pain modulation may explain individual differences in pain susceptibility, and duloxetine is proposed to control pain by increasing 5-HT/NA-mediated effects in descending pain modulation—the brain has been largely disregarded in the study of CIPN.

While numerous clinical trials have explored various agents with the potential to modify the development of CIPN by targeting its underlying mechanisms, no sufficient evidence is available to recommend a specific pharmacologic intervention [41,44]. Instead, the guidelines of the American Society of Clinical Oncology (ASCO) and the European Society for Medical Oncology (ESMO) suggest regular assessment of CIPN development, with particular vigilance of high-risk patients, considering some predisposing factors, which may include the specific chemotherapy agents employed, cumulative dosages, and individual patient characteristics [41,44]. It is noteworthy that the latter remains understudied.

The current treatment options for CIPN are insufficient. Patients and healthcare providers face significant challenges in effectively managing this debilitating condition. Available treatments mainly focus on symptom relief, such as pain management, but do not address the underlying mechanisms of CIPN [45]. Additionally, these therapies have limited efficacy and come with their own side effects [45,46]. Although duloxetine is the only recommended agent against CIPN [43,47], tricyclic antidepressants and anti-seizure medications are also repurposed in the treatment of painful CIPN, based on established evidence and guidelines for managing different types of neuropathic pain [4,41]. However, ASCO currently makes no recommendations on these therapies, but ESMO supports their consideration for treatment [41,44].

Nonpharmacological treatments may also be useful but there is some disagreement regarding the effectiveness of these approaches. A recent systematic scoping review and expert consensus process offer clinical recommendations for preventing and treating CIPN through non-pharmacological interventions [48]. Exercise, acupuncture, massage therapy, and nutritional interventions are highlighted as potential interventions to alleviate CIPN [48,49]. Also, given the established advantages of exercise in managing chronic pain and cancer and considering its low risk of harm, promoting increased physical activity should be an integral component of CIPN management [50,51,52]. Furthermore, noteworthy work has been carried out in applying brain–computer interface techniques, such as neurofeedback, with promising results in the mitigation of CIPN symptoms [53,54].

## 5. Mechanisms of CIPN

Chemotherapy agents are designed to destroy cancer cells but can also damage healthy cells, including those at peripheral nerves [25]. There are six classes of chemotherapeutic agents most associated with CIPN: the platinum-based antineoplastics (particularly oxaliplatin [55] and cisplatin [56]), the vinca alkaloids (particularly vincristine [57]), the epothilones (ixabepilone [58,59]), the taxanes (paclitaxel [60], docetaxel [61]), the proteasome inhibitors (bortezomib [62]), and immunomodulatory drugs (thalidomide [63]).

Chemotherapeutic drugs damage the nervous system structures and cause CIPN using complex mechanisms. It is well established that higher doses of chemotherapeutic agents and the number of chemotherapy cycles increase the probabilities of nerve damage and manifestation of neuropathic symptoms [22]. 

Although the focus of this narrative review is not the peripheral mechanisms of CIPN, it should be mentioned that they are diverse and are under investigation both in animal models and in humans since they play a partial role in CIPN pathophysiology. Preclinical studies have provided valuable insights, with animal models playing a crucial role in identifying the specific peripheral nerve damage caused by the different chemotherapeutic agents. Collectively, these studies showed various mechanisms underlying the effects of chemotherapy on peripheral nerves including mitochondrial damage, neuroinflammation, increased neuronal excitability in neurons of the dorsal root ganglion (DRG), and disruption of the neuronal cytoskeletal architecture and axonal transport [25,64,65]. For a more comprehensive review, please refer to [66,67]. Research into the peripheral mechanisms of CIPN has yielded promising results in both clinical and preclinical studies. For example, the administration of acetyl-L-carnitine decreases mitochondrial dysfunctions and prevents paclitaxel-, oxaliplatin-, and bortezomib-induced peripheral neuropathy [68,69,70,71]. However, a randomized controlled trial enrolling 409 patients found that acetyl-l-carnitine aggravated CIPN [72,73]. A similar pattern was observed with pregabalin, a medication that effectively alleviated CIPN in rodent models by interacting with voltage-gated calcium ion channels [74,75], which are upregulated in DRG during CIPN [76,77]. However, a recent phase III randomized double-blind, placebo-controlled trial using oral gabapentin for the prevention of paclitaxel-induced CIPN showed that gabapentin may not be effective in the prevention of that condition [78]. Due to the lack of effective treatments for CIPN based on current understanding, research should shift to exploring new mechanisms. Understanding the mechanisms of CIPN can lead to the development of more effective methods for diagnosis, treatment, and prevention of this condition, ultimately reducing its significant impact on patients. 

In addition to the damage of peripheral structures, neuroinflammation and/or neuroplastic changes at the CNS also account for painful CIPN [79,80]. Central sensitization, involving increases in responses of spinal cord and brain neurons, is believed to also play a pivotal role in the chronic pain and sensory abnormalities characteristic of CIPN. Understanding the mechanisms of CIPN can help us find new treatments to mitigate the impact that CIPN [4]. Taking this into account, the next sections will focus on changes in brain areas, namely those that are involved in descending pain modulation.

### 5.1. The Brain of CIPN Patients

In the past few decades, brain-imaging studies revealed changes in brain areas in patients who have experienced prolonged pain, namely the limbic system, the part involved in behavioral and emotional responses [81,82]. Abnormalities in pain processing, as revealed by neuroimaging, rather than just peripheral nerve damage or inflammation, are believed to be significant contributors to other chronic pain conditions like osteoarthritis [83,84], trigeminal neuralgia [85], post-herpetic neuralgia [86], chronic low back pain [87], and diabetic neuropathy [88,89]. However, little is known about these mechanisms in CIPN. Despite differences in patient populations, study designs, and sample sizes, Table 1 presents the main findings of the only three neuroimaging studies that have assessed brain structure and function in patients with painful CIPN [90,91,92].

These studies applied fMRI to investigate brain activation patterns in response to chemotherapy treatments. Results reveal distinct patterns of brain activation associated with pain perception in CIPN patients, shedding light on the central mechanisms contributing to chronic neuropathic pain in this population. In 2015, Nudelman and his team conducted a study involving 45 women diagnosed with non-metastatic breast cancer, comparing brain scans before (baseline) and after (1 month and one year later) chemotherapy with women who had cancer but did not undergo chemotherapy [90]. At one-month post-chemotherapy, increased severity of CIPN was correlated with heightened perfusion in several brain regions, including the superior frontal gyrus, cingulate gyrus, left middle gyrus, and medial frontal gyrus. Additionally, at the one-year follow-up, an escalation in CIPN severity from the pre- to the one-month post-chemotherapy assessment was associated with increased perfusion in the left cingulate gyrus and left superior frontal gyrus. However, after one year, no significant associations were found between CIPN severity and brain perfusion [90]. Boland and colleagues also conducted a case-control study in which 24 participants, 12 with multiple myeloma (MM) and CIPN and 12 healthy volunteers, underwent fMRI during the application of noxious heat-pain stimulation [91]. In this study, patients with multiple myeloma exhibited greater activation in the left precuneus and lower activation in the right superior frontal gyrus for both foot and thigh compared to healthy volunteers. Also, activation in the left frontal operculum (near the insula) in response to heat-pain stimulation of the foot was associated with worse CIPN [91]. Recently, Seretny and collaborators conducted a prospective, multicenter cohort study using brain fMRI to study the development of chronic sensory/painful CIPN nine months after chemotherapy. A total of 12 out of 20 patients, classified as CIPN positive (CIPNþ), demonstrated robust activity in sensory, motor, attentional, and affective brain regions in response to punctate stimulation. Additionally, a region-of-interest analysis focusing on the periaqueductal grey (PAG) matter, hypothesized to be relevant for developing CIPNþ, showed significantly increased responses in CIPN-negative (CIPNe) compared to CIPNþ patients [92].

Collectively, the imaging studies in patients with CIPN indicate that this condition is accompanied by structural and functional changes in the brain. The causality relations cannot be established, namely due to the design of the studies and the lack of tools to perform neurochemical characterization of the involved brain regions.

### 5.2. Contribution of Preclinical Research 

In this section, we will review the evidence obtained in animal models that may explain the role of pain modulation from the brain in the appearance of pain during CIPN. Although the features of CIPN models need to be critically reappraised [93], several studies focused on descending pain modulation, using the neurochemical systems targeted by duloxetine, namely 5-HT/NA. Recent imaging studies of CIPN models were performed [80,94,95,96], and this is an approach with potential translational perspectives.

The descending pain modulatory system is an intrinsic network of brain areas that exerts a bidirectional (inhibition/facilitation) control of the nociceptive transmission from the spinal cord [10,11,12]. The PAG plays a key role in top-down pain modulation. It has reciprocal projections with the prefrontal cortex (PFC), anterior cingulate cortex (ACC), and amygdala, along with several regions of the thalamus, hypothalamus [97,98,99], and brainstem regions, such as the locus coeruleus (LC), the rostral ventromedial medulla (RVM), and areas of the medullary reticular formation [100,101,102,103]. The PAG relays its descending input through the RVM since it does not send direct projections to the spinal cord [104]. The RVM is a brainstem center that exerts bidirectional control, leading to a balance of inhibition (antinociception) and facilitation (pronociception) of nociceptive transmission at the spinal cord [105]. The PAG and RVM also project to noradrenergic areas, namely LC, which project to the spinal cord [106].

**Figure 1:**
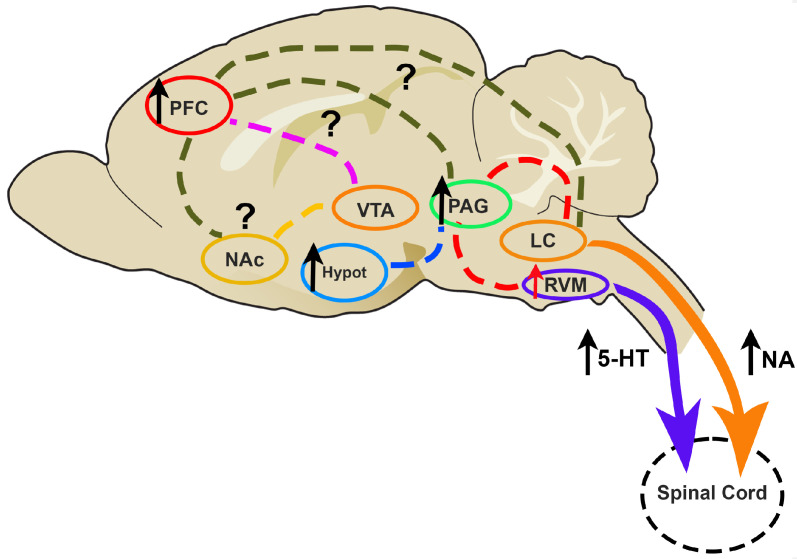
Brain mechanisms accounting for painful CIPN, according mainly to the results of preclinical studies. An increase in the activity of several areas leads to an imbalance of top-down pain modulation, towards facilitation, namely with increased release of -5-HT and NA at the spinal cord. The involvement of relevant circuits, such as the reward system, is under investigation. Abbreviations: Hypot: hypothalamus; LC: locus coeruleus; NAc: nucleus accumbens; PAG: periaqueductal grey; PFC: prefrontal cortex; RVM: rostroventromedial medulla; VTA: ventral tegmental area; purple arrow: descending serotoninergic pain modulation; orange arrow: descending noradrenergic pain modulation; different color lines: conections between brain areas; the symbol “?” shows neuronal circuits in which the effects of CIPN is not fully established.

It is noteworthy that descending pain modulation is also influenced by several corticolimbic regions. The connections between the ACC, PFC, and other mesolimbic areas, such as the ventral tegmental area (VTA) and nucleus accumbens (NAc), contribute to emotional, and cognitive control of pain perception and are involved in reward [107]. Describing these circuits and understanding their function in the context of neuropathic pain is now a focus of research across the field [108,109]. Figure 1 illustrates the brain areas that are affected by neuropathic pain in animal models treated with chemotherapy. There is still much to study, namely the connectivity between the brain areas involved in pain modulation.

The neurochemical modulation used in top-down circuits is complex [15], but the main neurotransmitters used are 5-HT, NA, and opioids. Though opioids display a key role in pain modulation and are used to treat painful conditions [13], this review will not focus on the role of opioids since they are not the first line of therapeutic approach to CIPN [44,110].

#### 5.2.1. New Approaches in Preclinical Studies of CIPN: Imaging Studies Using MRI and Spectroscopy Analysis

The difficulties in studying neuronal activation in the brain of rodent models with neuropathic pain are increasing. Traditionally, methods such as behavioral tests, electrophysiology, pharmacology, and molecular biology approaches have been used to examine the plasticity of the descending pain modulation system. Furthermore, neuroimaging is becoming an important technique for investigating brain activity in animal models and identifying local variations associated with pathological conditions such as chronic pain [111,112]. Neuroimaging techniques applied for brain imaging in pain research include electroencephalography (EEG), fMRI, manganese-enhanced MRI (MEMRI), and positron emission tomography (PET) [111]. These approaches have enabled the study of activation patterns in the brain using chronic pain models. The brain regions most frequently activated during persistent pain include frontal cortex areas, the hippocampus, amygdala, basal ganglia, and NAc [113,114,115,116].

The first studies on animal models of CIPN utilizing MRI methods appeared quite recently (Table 2). Research using a particular fMRI method based on water diffusion found that paclitaxel-induced neuropathy causes alterations in brain regions associated with the emotional and affective aspects of pain at the early stage of polyneuropathy [94]. Our group recently performed imaging studies using MEMRI that demonstrated that paclitaxel treatment increases the neuronal activation of the hypothalamus and PAG at a late stage of CIPN [80]. Besides these brain functional alterations, ex vivo spectroscopy revealed a significant metabolic change in brain regions comprising pain modulation at early and late stages of paclitaxel-induced neuropathy [80]. These alterations in supraspinal metabolites suggest that supplementary processes involving glial cells may be occurring during paclitaxel-induced neuropathy. Similarly, preclinical studies with non-human primates demonstrated the use of fMRI in the investigation of CIPN. Oxaliplatin-treated macaques presented higher activation of the secondary somatosensory and insular cortex in response to cold stimuli, which were attenuated by 5-HT/NA reuptake inhibitor, duloxetine [95,96].

The functional significance of these changes in brain activity and metabolism needs to be checked, particularly the connectivity between these brain regions and the involvement of neuroinflammatory events. Furthermore, it is crucial to thoroughly examine the impact of each chemotherapeutic agent on the brain during the progression of polyneuropathy, at several timepoints. This may be achieved by utilizing multiparametric MRI techniques (e.g., fMRI, MEMRI, magnetization transfer imaging), which will offer valuable insights into the underlying processes of CIPN and may allow to develop protocols that may be used to approach CIPN in its early phases.

#### 5.2.2. The Study of the Descending Pain Modulation during CIPN: Neurochemical Studies of the Serotoninergic and Noradrenergic Systems

Neuroplastic alterations in the descending modulatory pathways have been observed in both animal models of neuropathic pain and in humans [14]. These changes disrupt the balance between descending modulatory circuits promoting descending facilitation over inhibition, which may contribute to the persistence and installation of pain [15,16]. Clinically, patients with CIPN are treated with antidepressants, which boost monoamine levels [7]. Duloxetine, a 5-HT/NA reuptake inhibitor, is the only effective pharmacological treatment in alleviating pain during CIPN [117]. However, the exact mechanisms driving these changes in neurochemical systems remain largely unclear (Table 3).

##### Involvement of 5-HT in Descending Pain Modulation 

The 5-HT, a monoaminergic neurotransmitter, is synthesized from tryptophan through the action of the enzyme tryptophan hydroxylase [129]. Spinal 5-HT can exert both pro- and antinociceptive effects, depending on the specific subtype of the 5-HT receptor [130,131,132]. It is widely recognized that activation of the spinal receptors 5-HT1A and 5-HT7, coupled to Gi/o protein, suppresses nociception, while the activation of the receptors 5-HT2, coupled to Gq/11 protein, and 5-HT3, linked to non-selective cationic channel, has the opposite effect [133]. The proper functioning of the descending serotoninergic pain system is essential for maintaining the balance between facilitation and inhibition in the modulation of nociceptive transmission. The data suggest that chronic pain alters the descending serotoninergic modulatory pathway, with an imbalance favoring facilitation, which might explain how chronic pain develops and is maintained [15,16]. The depletion of serotoninergic neurons in the RVM or serotoninergic pathways has been shown to reduce pain-like behaviors [134] and prevent sensory hypersensitivity in animal models of neuropathic pain [135]. Moreover, in several neuropathic pain models, the descending pain modulation system seems to adapt to persistent pain by enhancing the serotoninergic input to the spinal cord [136,137].

Our lab has developed pioneer work in what concerns paclitaxel-induced neuropathic pain. We showed that during CIPN, the RVM undergoes neuroplastic alterations including local increases in neuronal activation, particularly 5-HT neurons [118]. The increased activation of 5-HT neurons could be due to the activation of the p38MAPK pathway in the RVM [119]. Recently, new evidence about the activation of the RVM during CIPN was provided, namely by showing excitatory projections from PAG somatostatin neurons, which were proposed to activate RVM neurons, promoting descending pain facilitation [138].

Considering the overactivity of the serotoninergic RVM neurons, preclinical studies have shown an increased engagement of the descending pain modulation during CIPN [118,119]. Furthermore, paclitaxel-induced neuropathy is associated with increased levels of 5-HT in the spinal cord [118]. On the contrary, decreased levels of the spinal 5-HT decreased after oxaliplatin and cisplatin treatments [120,121]. Although we could conclude that other studies showed the involvement of 5-HT in descending pain, facilitation during CIPN may depend on the chemotherapeutic agent. Considering that there are some concerns about experimental reports in CIPN models [93,139], the studies need to be continued to fully understand the mechanisms subserving the descending serotoninergic pain modulation.

It is well established in several pain models that spinal 5-HT may induce pro-or antinociception effects depending on the target receptor subtype [133]. A study showed that the levels of 5-HT1A receptor mRNA were downregulated in the spinal dorsal horn after oxaliplatin treatment [122,123]. It was also reported, with greater evidence, that its mRNA level rose in the rodent spinal dorsal horn and that treatment with a 5-HT1A agonist inhibited the oxaliplatin-induced pain-like behaviors [123,140,141,142]. Curiously, no changes were reported in spinal 5-HT1A expression after paclitaxel and vincristine treatment [123]. Regarding 5-HT2 receptors, the information available about its role in chemotherapy-induced neuropathic pain is not very clear. In a model of oxaliplatin-induced neuropathy, a 5-HT2A receptor antagonist was shown to suppress the analgesic effects of neurotrophin on nociceptive behaviors [143]. Moreover, 5-HT2A receptor knock-out mice did not exhibit any vincristine-related painful phenotype, suggesting that the 5-HT2A receptor could be involved in vincristine-induced neuropathic pain. In addition to these modifications, vincristine treatment also triggered the overexpression of this receptor in the superficial layers of the spinal dorsal horn [124]. It is noteworthy that oxaliplatin also induced an increase in the 5-HT2C expression in the spinal cord and its antagonism produced antinociception [125].

The 5-HT3 receptor, which is the only ionotropic 5-HT receptor with excitatory functions, has a pronociceptive role in numerous models of chronic pain, including during CIPN [118,119,126,144,145]. Our group showed that spinal administration of 5-HT3A receptor antagonist induced antinociception in paclitaxel-treated animals [118]. Likewise, Liu et al. confirmed that the intrathecal injection of the 5-HT3A receptor antagonist partially reversed the paclitaxel-induced neuropathic pain [119]. Furthermore, our work showed that the antinociceptive effects of the 5-HT3A receptor antagonist are associated with the overexpression of this receptor in the superficial dorsal horn [118]. Moreover, recent work also showed that the spinal 5-HT3A receptor seems to be a perfect target for natural products, such as natural coumarin, to treat vincristine-induced neuropathic pain [126].

There is a clear gap of knowledge on the contribution of each 5-HT receptor in pain installation and management during CIPN. It will be critical to explore the participation of other l 5-HT receptors in the spinal cord during CIPN, as their role and expression seem to be dependent on the chemotherapeutic drug and the stage of CIPN.

##### Involvement of NA in Descending Pain Modulation 

The biosynthesis of NA involves the enzymes tyrosine hydroxylase (TH) and dopamine-β-hydroxylase (DBH), which are frequently used as surrogates of noradrenergic pathways [146].

Descending noradrenergic projections originate from three brainstem clusters of noradrenergic neurons: the A6 (the LC and nucleus subcoeruleus), as well as the A5 and A7 noradrenergic cell groups [147,148,149,150,151,152]. These fibers release NA at the spinal cord, where it inhibits peripheral input and spinal dorsal horn neurons by activating α2-adrenergic receptors (α2-AR) [146].

Alpha-adrenergic receptors (AR) are divided into α1- (subtypes 1A, 1B, and 2D) and α2- (subtypes 2A and 2C) AR, which are part of the G protein-coupled receptor family [146,153]. In the DRG and spinal dorsal horn, the α1-AR are combined to phospholipase C via Gq protein or directly to calcium influx facilitating nociceptive transmission [154]. Otherwise, the α2-AR are combined to adenylcyclase via Gi protein, reducing the formation of cyclic AMP and calcium influx during action potential to inhibit neurotransmitter release [146]. These α2-AR are located in noradrenergic terminals (as autoreceptors), spinal neurons, central terminals of peripheral nerve fibers, and DRG neurons [146,155]. When NA is released from descending fibers, it produces analgesia by acting on distinct subtypes of spinal α2-AR. The α2A-AR subtype is highly expressed in descending noradrenergic fibers, and its activation inhibits NA release to spinal neurons [153]. This subtype is also found on central terminals of primary afferents that contain substance P and glutamate; its activation leads to presynaptic inhibition of these neurotransmitters, promoting antinociception [156]. The α2C-AR is located on DRG terminals and postsynaptically on spinal excitatory interneurons. Activation of postsynaptic α2C-AR inhibits transmission of the nociceptive signals to the supraspinal pain processing regions [157].

Chronic pain induced neuroplastic modifications at the descending noradrenergic modulation, which may promote the chronification of this condition [158,159]. 

It is well established that NA release at the spinal cord mainly comes from the pontine LC [146] and induces analgesia by activating spinal α2A-AR, which inhibits nociceptive transmission in the spinal dorsal horn [160]. 

Some studies have reported a diminished role of the noradrenergic system in animal models of neuropathic pain [158,161]. However, this view is not universally accepted, as other studies suggest that the descending noradrenergic system activity may increase to counteract the enhanced nociceptive input from damaged peripheral fibers in traumatic neuropathic pain models [162,163]. The impact of changes in descending noradrenergic pain modulation during CIPN is still largely unexplored (Table 3). Recent findings have shed some light on this, demonstrating that paclitaxel treatment increases TH levels in the LC [127]. Moreover, in paclitaxel-induced neuropathy, there is an engagement of descending noradrenergic inhibition. Reboxetine, a selective NA reuptake inhibitor, has been shown to produce antinociceptive effects in paclitaxel-treated rats [128]. This animal model also exhibited increased expression of DBH in the spinal cord [128]. Supporting those results, the increased levels of NA in the spinal cord were also reported in paclitaxel-induced neuropathy [127]. Noradrenaline inhibits the nociceptive transmission by activating spinal α2-AR located both pre- and postsynaptically [156,160,164]. The results of our investigation demonstrated that the behavioral effects of spinal injection of an α2-AR agonist or antagonist were significantly stronger in the paclitaxel group. But paclitaxel did not change α2-AR expression, suggesting an increased potency of the spinal α2-AR [128]. On the contrary, another study showed that paclitaxel induced an increase in spinal α1- and α2-AR gene expression [127]. Similarly, a study using oxaliplatin-injected animals indicated that spinal administration of NA yields an inhibitory effect in spinal hyperactivation and nociceptive behaviors [165].

As discussed for the descending serotonergic modulation of pain, it is necessary to better understand the descending noradrenergic pain modulation in CIPN, specifically the mechanistic differences and similarities that exist in different animal models, since the descending noradrenergic inhibitory controls seem to be crucial for pain relief by supraspinal drugs during CIPN [166]. 

##### Other Neurochemical Systems

Cannabinoids (CB) are compounds that possess pain-relieving qualities, and the endocannabinoid system appears to have an important function in the regulation of pain through descending modulation [167,168,169]. Multiple cannabinoid receptors have been identified, namely the CB1 cannabinoid (CB1) receptor and the CB2 cannabinoid (CB2) receptor. Both receptors are G protein-coupled receptors with inhibitory effects of adenylyl cyclase and certain calcium and potassium channels. They also activate the mitogen-activated protein kinase [170]. It is noteworthy that CB1 receptors can also activate or arrest various G-proteins, as well as form heterodimers with opioids or serotonin receptors [171,172,173]. These characteristics render CB receptors a compelling focus for therapeutic intervention. The CB1 receptors are expressed in several areas involved in pain modulation, such as neurons of the cerebral cortex, basal ganglia, amygdala, PAG, hypothalamus, brainstem medullary nuclei, and spinal cord [174], but they are also expressed by astrocytes [175]. The CB2 receptors are expressed in neurons of the brainstem, cerebellum, basal ganglia, PFC, and hippocampus [176], but also in microglia, astrocytes, and oligodendrocytes [177,178,179]. There are studies indicating that prolonged pain conditions lead to an overregulation of CB2 receptors in both the peripheral and CNS [180,181,182]. A recent publication showed that the peripheral CB2 receptor appears to have a role in suppressing paclitaxel-induced neuropathic pain [183]. Pain-relieving properties of cannabis in CIPN have been described. Combined or alone, cannabidiol (CBD) and tetrahydrocannabinol (THC) seem to be effective in attenuating pain-like behaviors in paclitaxel-, oxaliplatin- and vincristine-induced neuropathic pain [184]. It was also reported that the CB1 and/or CB2 receptor agonists also present analgesic effects in paclitaxel-, cisplatin- and vincristine-induced neuropathy [185,186,187]. Moreover, paclitaxel induced the expression of CB2 receptors in spinal dorsal horn microglia, which was attenuated by a selective CB2 agonist [188]. Furthermore, the interaction of CB receptors with other receptors, e.g., opioid receptors, has been described in CIPN with a potential role for therapeutic targets. Combined CB1 receptor and delta-opioid receptor agonists reversed the paclitaxel-induced hypersensitivity through the spinal upregulated CB1 receptor/delta-opioid receptor heteromers [189].

While there is ongoing research on the use of cannabis to treat CIPN, more extensive investigation is required to fully understand the processes of action of CB, as well as the synergistic interactions of CB receptors with other receptors, namely opioid receptors, and the specific alterations that take place in brain areas involved in endocannabinoid modulation.

The implication of the cholinergic system in changes in pain modulation during chronic pain was demonstrated with a loss of tonic spinal inhibition of nociceptive transmission in neuropathic pain models [190]. The cholinergic system involves two different types of receptors: the nicotinic acetylcholine receptor (nAChR) and muscarinic acetylcholine receptor (mAChR) [191]. 

The nAChRs, ionotropic receptors, present a wide range of subunits (α1–α10, β1–β4, γ, δ, and ε), which can combine to form different subtypes of this receptor [192]. The metabotropic mAChRs present distinctive types of receptors: m1AChR, m2AChR, m3AChR, m4AChR, and m5AChR. m1 AChR, m3AChR, and m5AChR are excitatory, and m2AChR and m4AChR are inhibitory [193]. The nAChRs and mAChRs regulate the pain modulation at the spinal cord and at supraspinal levels. For example, it is well established that nAChR may stimulate descending inhibitory pathways at the supraspinal areas, via spinal α2-Ars and 5-HT3 receptors [194]. For an extensive review, see [191].

A large body of evidence indicates that the cholinergic system may also play a role in the onset and progression of painful CIPN [195,196]. Pharmacological interventions that increased the quantity of acetylcholine (ACh) in the synaptic cleft, such as Ach inhibitors, inhibitors of Ach exocytosis (botulinum neurotoxin A), and a precursor of choline (citicoline), exhibited antinociceptive effects on mechanical and/or thermal hypersensitivity in rodent models of CIPN [195,196,197,198]. Furthermore, nicotine (nAChR agonist) also demonstrated analgesic effects and could prevent mechanical and thermal hypersensitivity in paclitaxel-treated rats [199]. In addition, other more specific nAChR agonists, namely compounds that bind to the α4β2 nAChRs seem to be more effective in the resolution of the mechanical hypersensitivity induced by vincristine and oxaliplatin [200,201]. Likewise, α7 nAChRs agonists presented an analgesic effect in pain-like behaviors in rodents treated with oxaliplatin and paclitaxel [202,203,204]. There is some evidence that systemic α9/α10 nAChRs antagonists present antinociceptive effects in both mechanical and/or thermal sensorial modalities in rodent models of oxaliplatin- and paclitaxel-induced neuropathy [205,206,207,208,209,210,211,212,213]. Surprisingly, m1AChRs agonists and antagonists reversed mechanical and thermal hypersensitivity induced by paclitaxel and oxaliplatin [214,215]. Noteworthy, spinal m2AChRs antagonist, methoctramine, reversed the analgesic effect of donepezil, a cholinesterase inhibitor, only in paclitaxel-treated animals [196].

Although the cholinergic system is well studied in pain modulation, the role of this neurochemical system in CIPN requires more investigation, particularly at the supraspinal level, to discover new therapeutic options.

##### Pharmacological Interventions

Given the change in pain modulatory areas and the significant role of the brain in the pathophysiology of CIPN, it is crucial to review brain interventions in animal models of CIPN to enhance our understanding of the pathology.

For several years, efforts have been made to evaluate the effects of specific agonists, antagonists, and repurposed drugs administered directly into the brain, to improve CIPN symptoms. Preclinical studies using paclitaxel-treated rats or mice have shown that the intracerebroventricular administration of selective kinin B1 and B2 receptor antagonists, gabapentin (voltage-gated calcium channel inhibitor), and a novel N-type voltage-gated calcium channel blocker resulted in analgesic effects for thermal and/or mechanical hypersensitivity [166,216,217].

Reasonably better studied are the effects of brain interventions in CIPN using oxaliplatin-induced neuropathy animal models. Oxaliplatin-treated macaques exhibited an improvement in cold hypersensitivity following the administration of the GABA_A_ receptor agonist, muscimol, into the secondary somatosensorial cortex and insula [95]. Additionally, muscimol injection into the dorsolateral PAG induced analgesia in oxaliplatin-treated rats [218]. Local administration of interleukin 1β and 6, and tumor necrosis factor α antagonists in the same brainstem area also ameliorated CIPN symptoms [218]. Moreover, the injection of m2AChR agonist, oxotremorine, into the posterior insula reversed the mechanical allodynia in oxaliplatin-treated rats [195]. Similarly, triple monoamine reuptake inhibitors, a selective NA and 5-HT reuptake inhibitor, and a selective 5-HT reuptake inhibitor administrated directly into the ACC reversed different pain-like behaviors in oxaliplatin-treated mice [219]. 

Other brain interventions, specifically the intracerebroventricular administration of CDP-choline (which increases choline and Ach), a selective Gi/o protein inhibitor, a GIRK1 channel blocker, a PKC inhibitor, and orexin-A also induced analgesia in oxaliplatin-treated rodents [197,220,221,222,223].

While brain interventions hold promise for alleviating the effects of painful CIPN, further preclinical studies are necessary. These studies should involve other chemotherapy drugs and take advantage of PET-MRI neuroimaging techniques, which enable the correlation of drug-induced changes in metabolism and neurotransmission with structural and connectivity alterations. Additionally, although this section addresses pharmacological interventions with precise molecular targets, it should be noted that the role of natural compounds with therapeutic potential should not be discarded (reviewed in [224]) but it is not the focus of the current review.

## 6. Concluding Remarks and Future Perspectives

This narrative review proposes that understanding the central mechanisms of CIPN involving the brain’s role in pain processing and response to chemotherapy can leverage the approach to managing and potentially preventing this condition.

Preclinical studies, using animal models of CIPN, have attempted to contribute to a better understanding of the brain mechanisms underlying painful CIPN. Cutting-edge studies are starting to uncover functional and metabolic brain changes, namely in the pain modulation areas, in response to chemotherapy [80]. Furthermore, there is also a great effort to identify blood biomarkers for painful CIPN to use them in clinical practice [225]. These techniques have a good potential for translation to the patient’s treatment.

**Figure 2:**
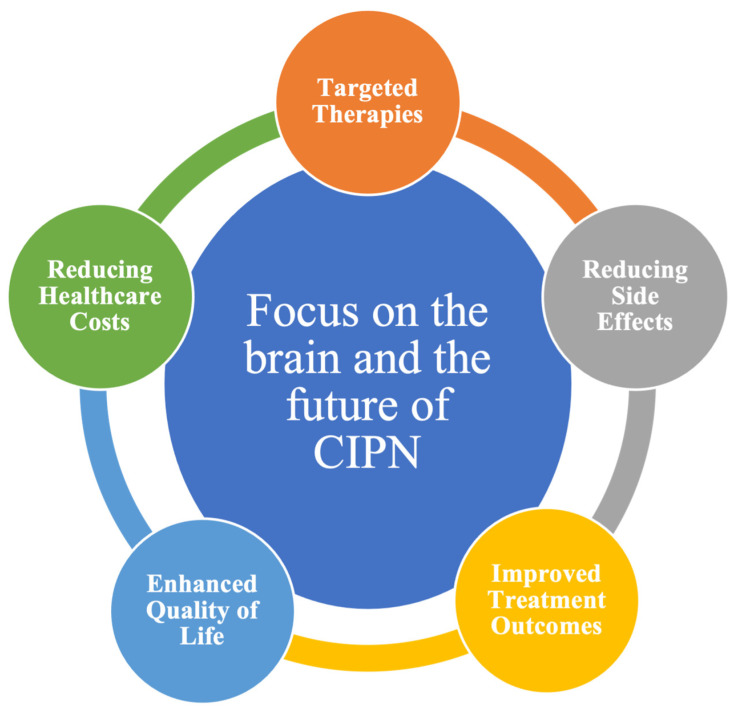
Brain’s role in CIPN and personalized chemotherapy.

In clinical settings, future challenges can be overcome with this new perspective of the CIPN approach (Figure 2). The main points are:

- Targeted Therapies: Understanding how the brain processes pain and responds to chemotherapy allows for developing treatments targeting these specific mechanisms. This means that medications and interventions can be tailored to the individual, addressing their unique pain processing and tolerance;
- Reducing Side Effects: A personalized approach based on a patient’s brain responses can reduce the risk of CIPN. By selecting prophylactic treatments, it is possible to mitigate or prevent painful CIPN;
- Improved Treatment Outcomes: The ability to reduce side effects based on an individual’s brain profile can improve treatment outcomes. By avoiding or minimizing CIPN, patients may be more likely to complete their prescribed chemotherapy regimens, leading to improved cancer treatment success;
- Enhanced Quality of Life: CIPN can have a profound impact on a patient’s quality of life, as it often leads to chronic pain and limitations in daily activities. Personalized treatment that minimizes the risk of CIPN can contribute to a better quality of life during and after cancer treatment;
- Reducing Healthcare Costs: Effective personalized treatment of CIPN can potentially reduce healthcare costs associated with treating CIPN-related complications, including pain management and rehabilitative care.

Shifting research focus on CIPN from the peripheral nerves to the CNS, particularly the brain, may reveal novel insights into the mechanisms of development and persistence of this neuropathic pain. Understanding the central mechanisms of CIPN could lead to more effective treatments and personalized approaches. Additionally, the personalized treatment of neuropathic pain has been widely discussed [226,227]. Adopting a multidimensional strategy in clinical settings that allows patient categorization based on central functional and structural biomarkers obtained from neuroimaging, together with clinical indicators such as patient-reported outcomes and sensory phenotyping seems to be the pathway for preventing and treatment of CIPN.

In conclusion, understanding the key role of the brain and personalizing treatment for painful CIPN based on individual brain responses offer the possibility of providing relief and improving the quality of life for cancer survivors.

## Figures and Tables

**Table 1:** Main findings of the three clinical human studies that have assessed brain plasticity using functional magnetic resonance (fMRI) in CIPN.

Study Design	Type of Chemotherapy	Effects of CIPN in Brain	Ref.
Longitudinal study: 24 women with chemotherapy, 23 women no chemotherapy	Combinations of paclitaxel, docetaxel, carboplatin, and cisplatin	↑ perfusion in the CG and SFG	[90]
Case-control study: 12 patients CIPN 12 healthy volunteers	Bortezomib, thalidomide, or vincristine	↑ activation in the precuneus ↓ activation in the SFG Activation in the FO associated with worse CIPN.	[91]
Prospective, multicenter cohort study: 20 patients	Bortezomib, oxaliplatin, paclitaxel, docetaxel, cisplatin	Prior chemotherapy (punctate stimuli): ↑ activity in insula, somatosensory cortex, thalamus and cerebellum in CIPNþ. ↑ activity of PAG in CIPNe	[92]

CG: cingulate gyrus; FO: frontal operculum; SFG: superior frontal gyrus; CIPNþ: CIPN positive; CIPNe: CIPN negative; ↑ increase; ↓ decrease.

**Table 2:** Main findings of the preclinical studies that have assessed brain plasticity using MRI techniques in CIPN.

Neuroimaging Approach	Species (Sex)	CIPN Model	Main Results	Ref.
DW imaging—quantitative anisotropy	Rats (males)	Paclitaxel	Reorganization of gray matter in the PFC, amygdala, hippocampus, hypothalamus and striatum/NAc	[94]
Rs functional connectivity	Rats (males)	Paclitaxel	Altered connections to the PAG	[94]
MEMRI	Rats (males)	Paclitaxel	↑ activation of hypothalamus and PAG	[80]
Ex vivo spectroscopy	Rats (males)	Paclitaxel	Early CIPN: ↑ NAA levels in PFC ↑ NAA and lactate levels in hypothalamus Late CIPN: ↓ NAA levels in PFC ↑ taurine levels in PFC	[80]
fMRI	Non-human primates	Oxaliplatin	↑ activation of SSC and Insula	[95,96]

DW: diffusion weight; PAG: periaqueductal gray matter; PFC: prefrontal cortex; NAA: N-acetyl-aspartate; NAc: nucleus accumbens; SSC: secondary somatosensorial cortex; ↑ increase; ↓ decrease.

**Table 3:** Main findings of the preclinical studies that have assessed descending serotoninergic and noradrenergic pain modulation during CIPN.

Neurotransmitter System	CIPN Model	CNS Region	Main Results	Ref.
Serotoninergic	Paclitaxel	RVM	↑ 5-HT neuron activation	[118]
		SC	↑ 5-HT levels ↑ 5-HT3 receptors	
	Paclitaxel	RVM	↑ 5-HT neuron activation	[119]
	Oxaliplatin	SC	↓ 5-HT levels	[120]
	Cisplatin	SC	↓ 5-HT levels	[121]
	Oxaliplatin	SC	↓ 5-HT1A receptors	[122,123]
	Paclitaxel Vincristine	SC	↔ 5-HT1A receptors	[123]
	Vincristine	SC	↑ 5-HT2A receptors	[124]
	Oxaliplatin	SC	↑ 5-HT2C receptors	[125]
	Vincristine	FC Striatum Hippocampus	↑ 5-HT levels	[126]
Noradrenergic	Paclitaxel	LC	↑ TH expression	[127]
		SC	↑ NA levels ↑ α_1_-AR receptors ↑ α_2_-AR receptors	
		SC	↑ DBH expression ↑ α_2_-AR receptor potency	[128]

5-HT: serotonin; AR: adrenoreceptor; DBH: dopamine-β-hydroxylase; FC: frontal cortex; LC: locus coeruleus; NA: noradrenaline; RVM: rostroventromedial medulla; SC: spinal cord; TH: tyrosine hydroxylase; ↑ increase; ↓ decrease; ↔ unchanged.

## Data Availability

Since this is a comprehensive review, the availability of the data is not an issue to be considered.
